# Supplementary material for: A photoheterotrophic bacterium from Iceland has adapted its photosynthetic machinery to the long days of polar summer
Source: mSystems. 2024 Feb 20;9(3):e01311-23. doi: 10.1128/msystems.01311-23 (PMC10949492; doi:10.1128/msystems.01311-23)
Supplement: Supplemental material — Tables S1 to S3, Figures S1 to S7, and Text S1. [file msystems.01311-23-s0001.docx]

**Supplementary Material**

**A photoheterotrophic bacterium from Iceland has adapted its photosynthetic machinery to the long days of polar summer**

Jürgen Tomasch^1^, Karel Kopejtka^1^, Tomáš Bílý^2^, Zdenko Gardian^2^, Alastair T. Gardiner^1^, Sahana Shivaramu^1^, Michal Koblížek^1^, and David Kaftan^1,3,^*

*^1^Laboratory of Anoxygenic Phototrophs, Institute of Microbiology of the Czech Academy of Sciences, 37981 Třeboň, Czechia*

*^2^Institute of Parasitology, Biology Centre, Czech Academy of Sciences, 37005 České Budějovice, Czechia*

*^3^Department Chemistry, Faculty of Science, University of South Bohemia, 37005 České Budějovice, Czechia*

**Author for correspondence:* [*kaftan@alga.cz*](mailto:kaftan@alga.cz)

Keywords: AAP, gene expression, light adaptation, photosynthesis, Proteobacteria, *Sediminicoccus*

Running title: Phototrophic *Sediminicoccus* from Iceland

**This file contains:**

Supplementary Table S1 – S3.

Supplementary Figure S1 – S7.

Supplementary Text S1.

**Supplementary Table S1 - Primers and qPCR conditions.**

| **Target** | **Primer Name** | **Sequence (5’-3’)** | |
| --- | --- | --- | --- |
| LHU95_13885 | Sr_pufM_fw | CAGTTCATCAAGCTGCTGCC | |
|  | Sr_pufM_rv | CAATAGGCGTAGAAGGTGTGC | |
| LHU95_13995 | Sr_ppsR_fw | AGCTCGCTCTTTCTGGTCC | |
|  | Sr_ppsR_rv | CCTCATTCGGCAATTCGGC | |
| LHU95_05270 | Sr_rpoD_fw | AGAAATACACCAATCGCGGC | |
|  | Sr_rpoD_rv | CTCGATCATGTGGACCGGG | |
| **Step** | **Temperature [°C]** | **Time [s] Cycles** | |
| UDG activation | 50 | 120 Hold | |
| Initial denaturation | 95 | 180 Hold | |
| Denaturation | 95 | 20 | 40x |
| Annealing/extending | 61 | 60 |  |

**Supplementary Table S2 - General characteristics of the *Sediminicoccus* sp. KRV36 genome after annotation using NCBI PGAP.**

| **Attribute** | **Value** |
| --- | --- |
| Total bases | 4,911,255 |
| No. of chromosomes | 1 |
| Plasmids | none |
| GC content [%] | 68 |
| No. of RNAs | 58 |
| ncRNAs | 3 |
| tRNAs | 49 |
| Genes (total) | 4,660 |
| - CDS | 4,576 (98%) |
| - hypo. proteins | 84 (1.8%) |
| - pseudogenes | 26 (0.6%) |
| PGC length [kbp] | 41.4 |

Hypo., hypothetical; PGC, photosynthesis gene cluster

**Supplementary Table S3 – Presence of selected metabolic pathways in *Sediminicoccus* sp. KRV36 cells.**

| **Metabolic pathway** | **Presence/absence** |
| --- | --- |
| Energy Metabolism |  |
| TCA cycle | + |
| Gluconeogenesis/glycolysis | + |
| Oxidative phosphorylation | + |
| Autotrophic carbon fixation | + |
| Anoxygenic photosynthesis (*puf*M) | + |
| Nitrogen Metabolism |  |
| Nitrogen fixation (*nifH*) | ̶ |
| Dissimilatory nitrate reduction | ̶ |
| Assimilatory nitrate reduction | + |
| Denitrification | ̶ |
| Nitrification | ̶ |
| Urease (*ureC*) | + |
| Sulfur Metabolism |  |
| Sulfur oxidation (*sox*) | + |
| Sulfide oxidation (*sqr*) | + |
| Phosphorus Metabolism |  |
| C-P lyase (*phnJ*) | + |
| Iron Metabolism |  |
| Fe^3+^ transport using siderophores | + |
| Hemin uptake | + |
| Synthesis of storage compounds |  |
| Glycogen synthesis/breakdown | + |
| PHB synthesis/breakdown | + |
| Polyphosphate synthesis/breakdown | + |

+, enzyme/metabolic pathway present; ̶ , enzyme/metabolic
 pathway absent.


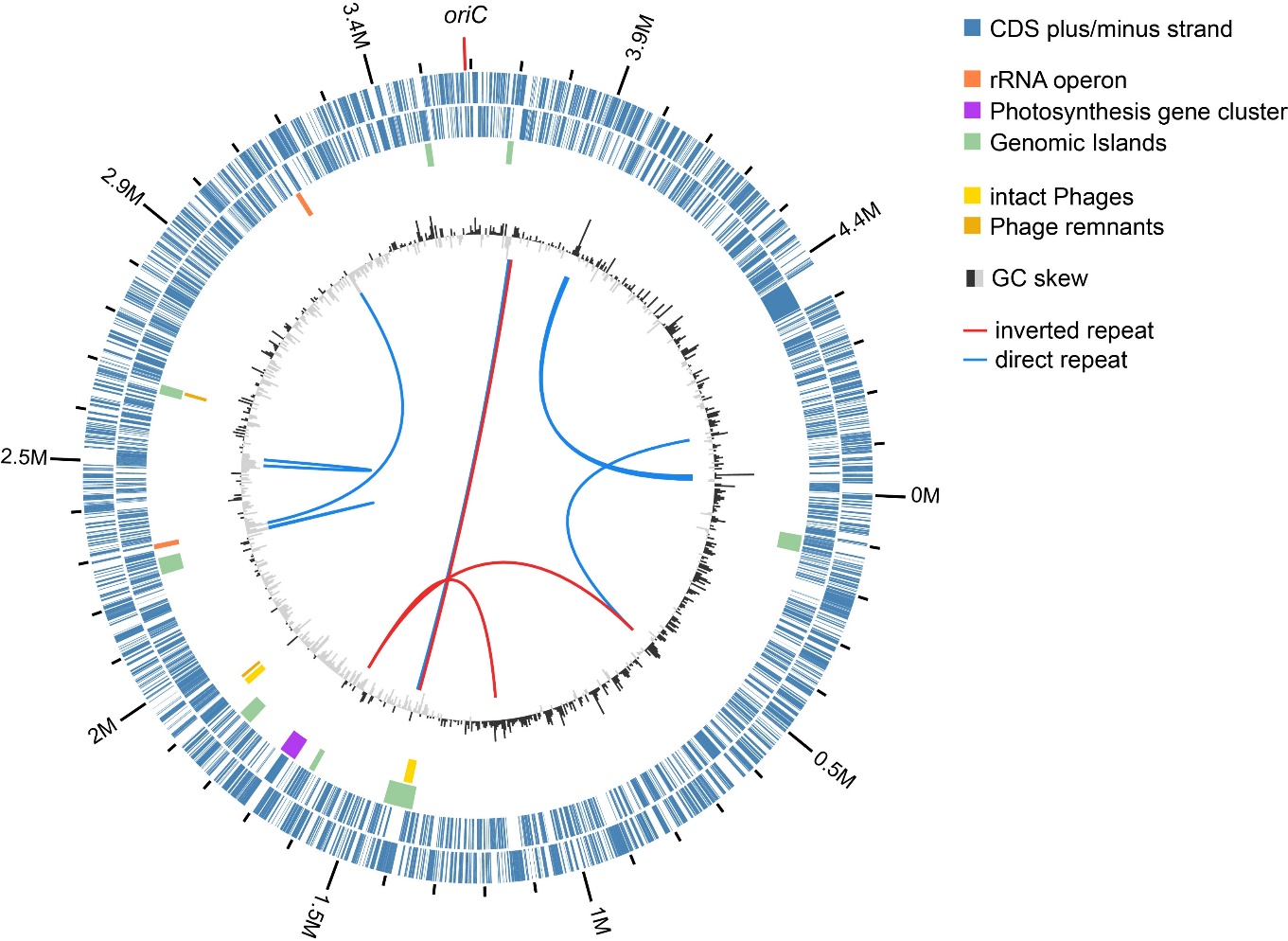


**Supplementary Figure S1 – Genome of *Sediminicoccus* sp. KRV36.** Single chromosome of 4.9 MB features inverted repeat regions, two of them are rRNA operons. Photosynthesis gene cluster is positioned unusually at the opposite site of the chromosome replication origin. The chromosome also contains two intact and two remnant phage genomes.


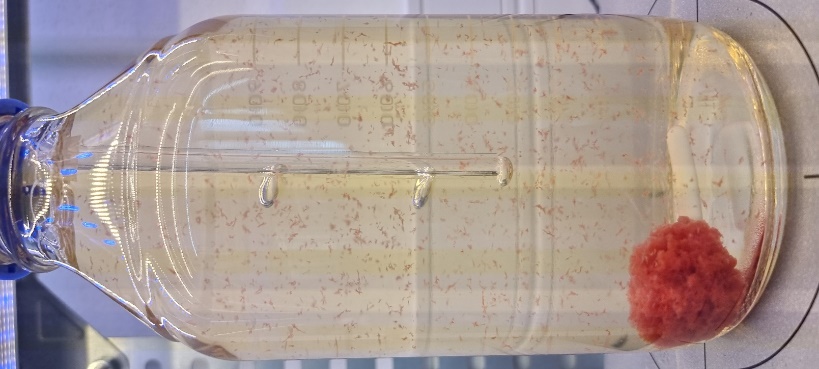


**Supplementary Figure S2 – Cluster of colonies of *Sediminicoccus* sp. KRV36.** Cells aerated by bubbling and stirring grown under constant light of 100 µmol *photon* m^-2^ s^-1^ aggregate to macroscopic colonies of several cm in diameter.

**A B**


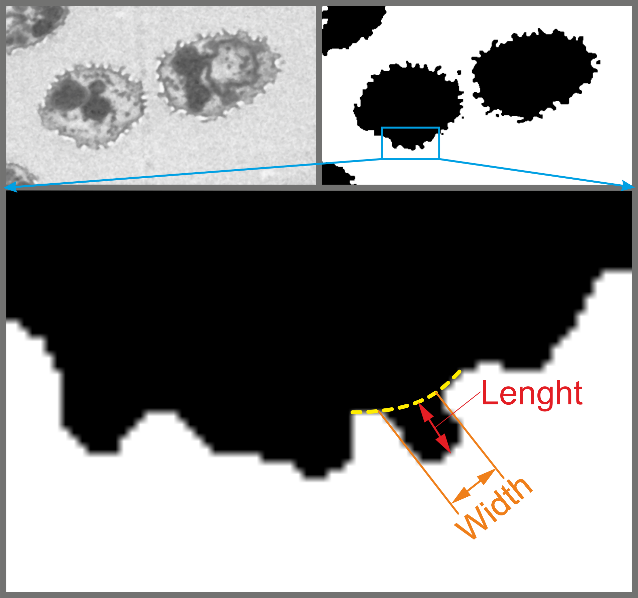

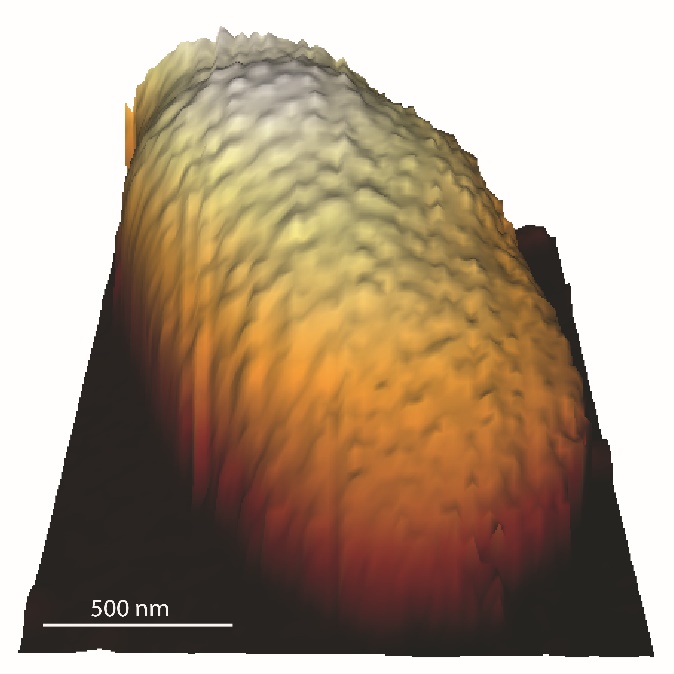


**Supplementary Figure S3 – Surface structures of *Sediminicoccus* sp. KRV36.** Imaging by EM (*A*), and AFM (*B*) shows outer layer of the cell wall is decorated by spikes. EM images were subjected to gradient segmentation and converted to a binary mask. Dimensions of the 49 individual features on the edge of several cells were then measured. The spikes were additionally imaged by AFM under physiological conditions.


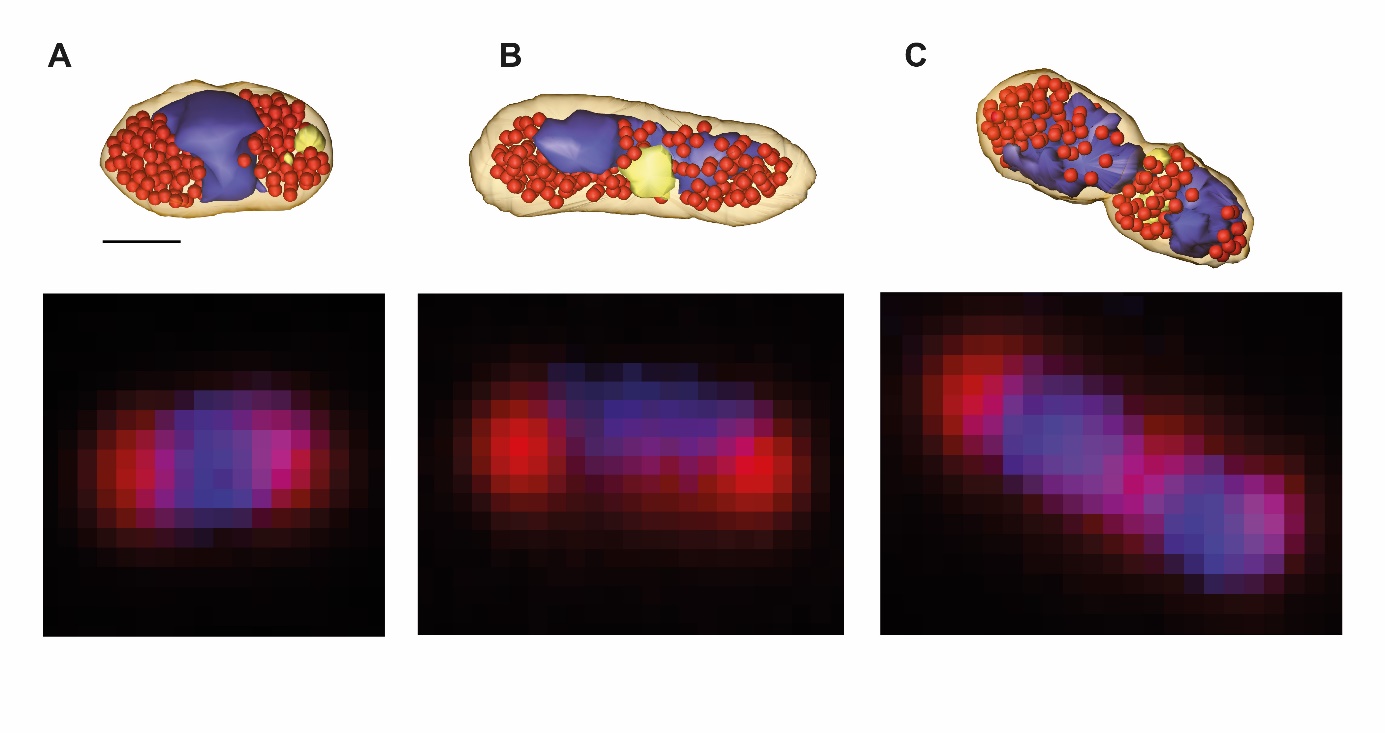


**Supplementary Figure S4 – Corelative imaging of *Sediminicoccus* sp. KRV36 cells using EM array tomography and fluorescence light microscopy.** Array tomography (SEM) (*Upper* panels) guided by fluorescence light microscopy (*Lower* panels) shows changes in cell ultrastructure on the course of cellular division. Small cell (*A*) exhibits a polar arrangement of chromatophores (red spheres) separated by a central DNA containing region (blue). Granules of storage metabolites (yellow) may contain polyhydroxybutyrate, polyphosphate or glycogen. A cell in an initial stage of cellular division (*B*) maintains chromatophores in a dominantly polar arrangement, yet chromatophores are already present in the vicinity of the future septum dividing the daughter cells. As the cell division progresses (*C*), the two still joined daughter cells have developed their own polar arrangements of chromatophores. Fluorescence light images were selected to match the EM tomograms. All images are scaled to the same dimensions, scale bar represents 500 nm.


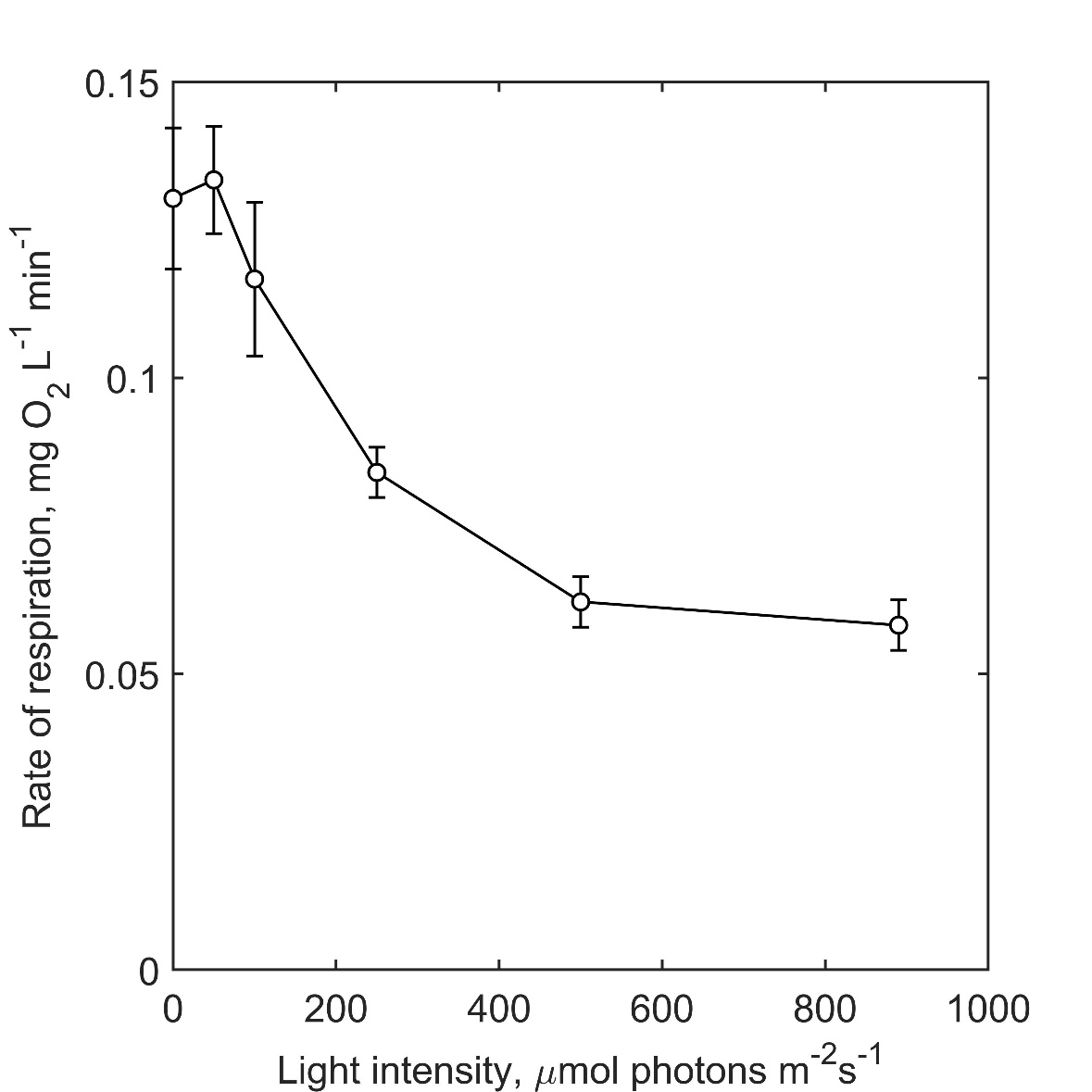


**Supplementary Figure S5 – Dependence of O_2_ respiration on light intensity.**


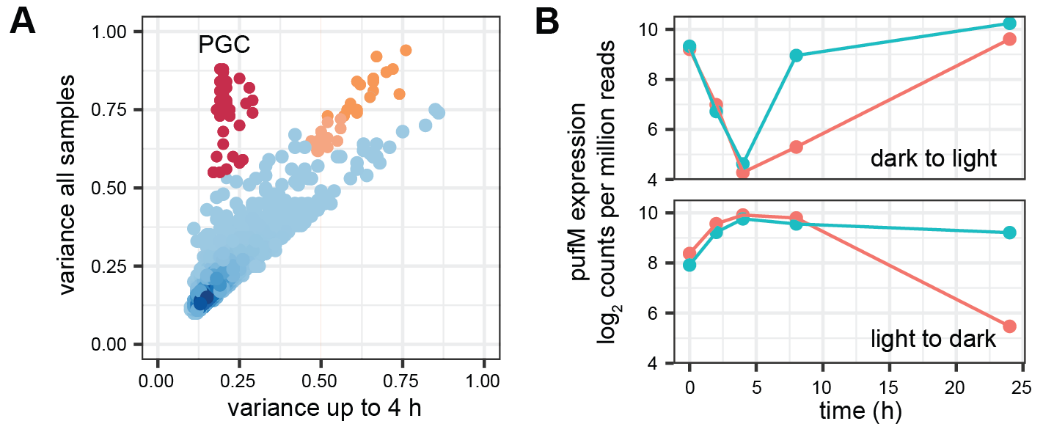


**Supplementary Figure S6 – Variance analysis of transcriptome data.** (*A*) Variance of expression for all time points is considerably higher for subsets of genes when compared to the early time-points, explaining the lack of significant changes in expression. (*B*) High variance is introduced by the different dynamics of the transcriptional response of mainly the PGC genes between the two biological replicate time series for each transition. Removing the time-points with highly variable samples resulted in a corrected assessment of statistical significance of gene expression changes. Note that the general trend of de-repression in the light is preserved between both time-series. For additional confirmation RT-qPCR has been performed for selected genes.


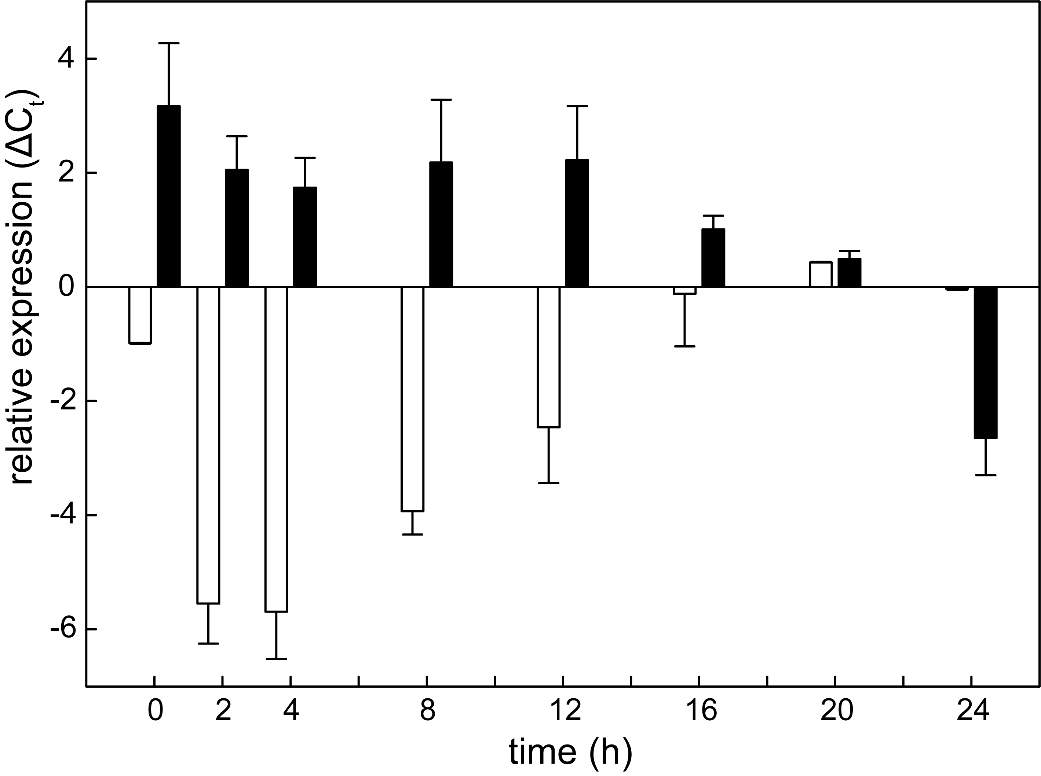


**Supplementary Figure S7 – Transcriptional response of the *ppsR* gene to changing light regimes.** White bars represent time series for the transition of dark-adapted cells to continuous-light regime, black bars represent a time series for the transition of light-adapted cells to continuous-dark regime. The ΔC_t_ mean values and standard deviations from two biological replicates are shown. C_t_, threshold cycle.

**Supplementary Text S1 – Genome based reconstruction of metabolic pathways**

The *S*. sp. KRV36 genome consists of one circular chromosome (4,911,255 bp) that contains two copies of rRNA operons (5S, 16S, 23S) with identical copies of the 16S rRNA gene (LHU95_RS16750 and LHU95_RS21345), 49 tRNA genes, and 4,576 protein-coding sequences (CDS). It also contained inverted repeat regions (two of which are rRNA operons) and four partly incomplete prophage regions. Overall, GC content was 68%. Genome characteristics are summarized in Supplementary Table S2.

*Energy and carbon metabolism.* The strain contains genes coding for all enzymes of the tricarboxylic acid cycle and gluconeogenesis/glycolysis (Embden-Meyerhof-Parnas) pathway and genes coding for protein complexes involved in oxidative phosphorylation. (Supplementary Table S6). None of the key genes coding for enzymes involved in autotrophic fixation (such as RuBisCO, ATP-citrate-lyase, or CO-dehydrogenase/acetyl-CoA-synthase), were found in the genome, in accordance with analysis of other AAPs (Yurkov and Beatty, 1998). The genome also possesses genes necessary for fully functional photosynthetic apparatus with RC-LH1 complexes (Supplementary Table S6) organized in a 41.4-kb-long photosynthesis gene cluster (PGC; LHU95_RS13810-LHU95_RS13995), positioned opposite of the predicted replication origin. The structure of the PGC was similar to both *Erythrobacter* and *Roseobacter* clades, with unique deviations. Notably, an additional gene (*hemC*; LHU95_RS13990) for heme synthesis was present, two regulators (genes *ppaA;* LHU95_RS13965 and *ppsR;* LHU95_RS13970) sensitive to light intensity and oxygen concentration were near the end of the PGC.

*Nitrogen metabolism.* Bacteria need nitrogen for processes such as nucleotide, amino acid, and vitamin synthesis, and energy generation. Common nitrogen sources include nitrate (NO_3_^-^), ammonia (NH_3_) and ammonium (NH_4_^+^), and organic compounds such as nucleic acids, proteins and peptides, and urea. Extracellular enzymes are often produced to break down complex organic compounds into smaller nitrogen-containing molecules that can be transported into the cells. We identified genes and gene operons coding for a nitrate/nitrite (LHU95_RS18810-LHU95_RS18820), ammonium (LHU95_RS00725), as well as for general (LHU95_RS07920-LHU95_RS07930) and branched (LHU95_RS08660-LHU95_RS08680) amino acids, and urea (LHU95_RS14120-LHU95_RS14140) transporters.

Bacteria are able to reduce nitrate through three main pathways: denitrification, dissimilatory nitrate reduction, and assimilatory nitrate reduction. Denitrifications results in release of N_2_. Other two pathways produce ammonium, which is bioavailable for other microorganisms. In KRV36, only the assimilatory nitrate reduction pathway is present (Supplementary Table S6). This is consistent with the data from physiological experiments done with the type strain R-30^T^ by Qu and colleagues (Qu *et al.*, 2013).

We did not identify genes coding neither for ammonia transporter(s) nor for nitrification enzymes. We found a gene operon (LHU95_RS02785-LHU95_RS02815) with genes coding for the enzyme urease (EC 3.5.1.5), potentially allowing the strain to utilize urea as a nitrogen source. Interestingly, Qu and colleagues (Qu *et al.*, 2013) stated that urease was absent in the type strain R-30^T^.

*Sulfur metabolism.* Bacteria can utilize sulfur both in reduced and oxidized forms (Dahl 2017; Wu *et al.*, 2021). Oxidation of reduced sulfur compounds results in an energy yield (Dahl 2017). Thiosulfate can be oxidized to sulfate using the sulfur oxidizing (Sox) complex (Friedrich 1997; Kelly *et al.*, 1997). We identified a complete *sox* gene cluster (Supplementary Table S6) in the genome, thus thiosulfate might be used a substrate to generate energy in the KRV36 strain. Moreover, we discovered a candidate gene (LHU95_RS09755) coding for the enzyme sulfide:quinone oxidoreductase (SQR). Oxidation of sulfide into elementar sulfur using SQR was documented for both anoxygenic phototrophic bacteria (Schütz *et al.*, 1997) and cyanobacteria (Arieli *et al.*, 1991).

*Phosphorus metabolism*. In freshwater environments phosphorus is a limiting nutrient (Elser *et al.*, 2007). Bacteria typically obtain it through the uptake of inorganic phosphate. We identified genes coding for a high-affinity inorganic phosphate acquisition system (*pstSCAB*; LHU95_RS02840-LHU95_RS02855) and a regulatory system (*phoUB*; LHU95_RS02860, LHU95_RS02865) which controls the expression of inorganic phosphate transport genes (Yuan *et al.*, 2006). Furthermore, we found all the genes necessary for the transport of methylphosphonate, an organophosphorus compound, and its cleavage (using the C-P lyase) to methane and phosphate (Supplementary Table S6).

*Iron metabolism.* KRV36 has a gene repertoire for transport of iron ions (Fe^3+^) chelated to siderophores, or bound to a heme-binding protein (hemin) (Supplementary Table S6). While siderophores transport free iron ions from environment into the cytoplasm, the hemin-uptake mechanism scavenges heme (a molecule containing iron) released from dead cells.

*Synthesis of vitamins.* We identified complete pathways for synthesis of thiamine (vit. B1), riboflavin (vit. B2), niacin (vit. B3), pyridoxin (vit. B6), and biotin (vit. B7).

*Storage compounds.* AAP bacteria deposit nutrients and energy in different compounds such as glycogen, polyhydroxybutyrate (PHB), and polyphosphate in form of granules in their cytoplasm. Glycogen is the main energy storage molecule in bacteria. It is a highly branched polysaccharide which can be quickly mobilized and converted into glucose and further metabolized. KRV36 genome contains an operon accommodating genes which encode enzymes responsible for glycogen synthesis and breakdown (Supplementary Table S6). PHB is a type of storage polymer that phototrophic bacteria can synthesize when they have access to excess carbon and are limited by nitrogen or phosphorus (Brandl *et al.*, 1991; Monroy and Buitrón, 2020). The resulting granules can be later metabolized and used as a carbon and energy source. We found all genes essential for both PHB anabolism and catabolism in the KRV36 genome (Supplementary Table S6). Polyphosphate is a linear polymer that can serve as a source of phosphorus and energy. Analysis of the KRV36 genome revealed genes coding for enzymes allowing to synthetize the polyphosphate granules and, when needed, also to utilize them (Supplementary Table S6).

**SUPPLEMENTARY REFERENCES**

Arieli, B., Padan, E., & Shahak, Y. (1991). Sulfide-induced sulfide-quinone reductase activity in thylakoids of Oscillatoria limnetica. *Journal of Biological Chemistry*, *266*(1), 104-111.

H. Brandl, R. A. Gross, R. W. Lenz, R. Lloyd, R. C. Fuller, The accumulation of poly(3-hydroxyalkanoates) in Rhodobacter sphaeroides. *Arch. Microbiol.* **155**, 337–340 (1991).

C. Dahl, “Sulfur Metabolism in Phototrophic Bacteria” in *Modern Topics in the Phototrophic Prokaryotes*, (Springer, Cham, 2017), pp. 27–66.

J. J. Elser, *et al.*, Global analysis of nitrogen and phosphorus limitation of primary producers in freshwater, marine and terrestrial ecosystems. *Ecology Letters* **10**, 1135–1142 (2007).

Friedrich CG (1997) Physiology and genetics of sulfur-oxidizing bacteria. Adv Microb Physiol 39:235-289

Kelly DP, Shergill JK, Lu WP, Wood AP (1997) Oxidative metabolism of inorganic sulfur compounds by bacteria. Antonie van Leeuwenhoek 1:95-107

I. Monroy, G. Buitrón, Production of polyhydroxybutyrate by pure and mixed cultures of purple non-sulfur bacteria: A review. *Journal of biotechnology* (2020) (May 4, 2023).

J.-H. Qu, *et al.*, Sediminicoccus rosea gen. nov. sp. nov., isolated from the sediment of a eutrophic lake. *J. Gen. Appl. Microbiol.* **59**, 463–468 (2013).

Schütz M, Shahak Y, Padan E, Hauska G (1997) Sulfide-quinone reductase from *Rhodobacter capsulatus* purification, cloning, and expression. J Biol Chem 272:9890-9894.

Wu, B., Liu, F., Fang, W., Yang, T., Chen, G. H., He, Z., & Wang, S. (2021). Microbial sulfur metabolism and environmental implications. *Science of The Total Environment*, *778*, 146085.

Z.-C. Yuan, R. Zaheer, T. M. Finan, Regulation and Properties of PstSCAB, a High-Affinity, High-Velocity Phosphate Transport System of Sinorhizobium meliloti. *Journal of Bacteriology* (2006) (May 3, 2023).

V. V. Yurkov, J. T. Beatty, Aerobic Anoxygenic Phototrophic Bacteria. *Microbiology and Molecular Biology Reviews* (1998) https:/doi.org/[10.1128/MMBR.62.3.695-724.1998](https://doi.org/10.1128/MMBR.62.3.695-724.1998) (January 6, 2023).
